# Unraveling the Molecular Basis of Stabilizing Selection by Experimental Evolution

**DOI:** 10.1093/gbe/evad220

**Published:** 2023-12-13

**Authors:** Christian Schlötterer

**Affiliations:** Institut für Populationsgenetik, Vetmeduni Vienna, Vienna, Austria

**Keywords:** sexual selection, stabilizing selection, polygenic adaptation, experimental evolution

## Abstract

Stabilizing selection provides a challenge to molecular population genetics. Although stabilizing selection is ubiquitous, its genomic signature is difficult to distinguish from demographic signals. Experimental evolution provides a promising approach to characterize genomic regions exposed to stabilizing selection. A recent experimental evolution study of *Aedes aegypti* populations evolving either with or without sexual selection found a pattern of genetic differentiation suggestive of relaxed stabilizing selection. I argue that this study could not have detected the signal of relaxed stabilizing selection. I highlight why incorrect statistical methods resulted in a high number of false positive candidate single nucleotide polymorphism (SNPs) and discuss the fallacy of functional validation of candidate SNPs for polygenic traits by RNA-mediated knockdown.

**Significance**

This commentary uses a recent study on sexual selection in *Aedes aegypti* to discuss the challenges of identifying genomic signatures of stabilizing selection. I also highlight that the large number of selection targets in this study are the result of an inadequate statistical test. Furthermore, the functional validation of candidates by RNA-mediated knockdown is undermined by the complex genetic architecture of the focal trait.

## Introduction

Most traits are subject to stabilizing selection. In the wake of the increasing number of genome-wide polymorphism data, many test statistics have been developed to detect the molecular signature of selection maintaining variation in a population (reviewed in Bitarello et al. 2023). However, like all polymorphism-based selection inference, these tests are highly sensitive to misspecifications of the demographic past (Bitarello et al. 2023). A further complication for the successful molecular characterization of stabilizing selection is the genomic architecture of a trait under stabilizing selection. Monogenic traits have the clearest selection signature as stabilizing selection on the trait is directly related to the underlying allele frequencies. In the case of polygenic traits, stabilizing selection is mediated by the joint effects of all loci with segregating variation contributing to the trait (Barton et al. 2017). This results in substantially more complex patterns and provides a hurdle to the molecular characterization of stabilizing selection.

Probably the most thorough understanding of allele frequency dynamics at polygenic traits under stabilizing selection comes from the analysis of shifts in trait optimum (Franssen et al. 2017; Jain and Stephan 2017; Höllinger et al. 2019). After an environmental shift, the contributing loci of a trait under stabilizing selection change in frequency. The first phase of adaptation in response to a shift in trait optimum closely resembles selective sweep dynamics, but when the new trait optimum is approached, the allele frequency change slows down (Hayward and Sella 2022). Remarkably, even when the trait optimum has been reached, the frequencies of contributing alleles continue to change with some alleles becoming fixed whereas others are lost (Franssen et al. 2017; Barghi et al. 2020; Hayward and Sella 2022). Although large effect loci are initially major drivers of the phenotypic change, at later phases, they are being replaced by loci of smaller effect (Hayward and Sella 2022). Ultimately, the population reaches an equilibrium between the emergence and spread of new contributing alleles and their loss, whereas the phenotype remains at the optimum.

Contrary to this well-studied case of a shift in trait optimum, very little is known about the dynamics of contributing alleles when the strength of stabilizing selection is reduced or even fully lost. A simple model assuming the complete loss of stabilizing selection predicts that contributing alleles are now governed by genetic drift, like neutral loci.

Experimental evolution provides a powerful experimental system to study the consequences of stabilizing selection and has been widely applied to characterize allele frequency changes after a shift in trait optimum (e. g. Burke et al. 2010; Orozco-terWengel et al. 2012; Burke et al. 2014; Griffin et al. 2017; Barghi et al. 2019). Recently, a study contrasted polymorphic *Aedes aegypti* populations with and without sexual selection and detected a genomic pattern that could be interpreted as the loss of stabilizing selection (Wyer et al. 2023): Populations evolved with sexual selection were genetically not diverged from each other or the ancestral population; monogamous populations were, however, diverged from each other and the ancestral population. Monogamous populations were less variable than populations with sexual selection. Here, I discuss why this pattern does not reflect the loss of stabilizing selection and why the statistical tests and functional validation used by Wyer et al. (2023) suffer from methodological problems. I propose that the data are much better explained by differences in effective population size between the two treatments caused by multiple mating of populations with sexual selection.

## The Experiment

Males of the mosquito *A. aegypti* are thought to be exposed to strong sexual selection. Wyer et al. (2023) were interested to identify genes involved in sexual selection. The central idea of their experiment was to use experimental evolution of replicated populations with two maintenance regimes, with and without sexual selection. Populations without sexual selection were more differentiated and lost more variation than populations with continued sexual selection. Wyer et al. (2023) interpret this pattern as the signature of relaxed selective constraint in the populations without sexual selection. Because populations with continued sexual selection were not differentiated from the ancestral population, this implicitly implies that stabilizing selection is operating. Based on the genomic signature the authors identified candidate loci and tested some of them functionally by dsRNA-mediated gene knockdown.

In the following, I will explain

1. Why the experimental design is not well-suited to detect loci subject to balancing selection
2. Why an inappropriate statistical testing resulted in an excess of false positives
3. Why the functional testing of candidate loci was not appropriate to confirm candidate loci
4. Why the results are better explained by multiple paternity in populations with sexual selection

## Expected Genomic Signature After Relaxed Selection

Two different scenarios for the genomic signature of sexual selection can be distinguished. The first scenario assumes that sexual selection, triggered by the competition between males, causes continued allele frequency changes of the involved genes. This scenario is supported by the high rate of evolution of male biased genes (Swanson and Vacquier 2002; Clark et al. 2006). Under the second scenario, sexual selection favors an optimal combination of allele frequencies in a population, a situation that is commonly called stabilizing selection, because frequency changes in both directions are deleterious.

The first scenario predicts that populations without selective constraints do not evolve (beyond drift) and populations with sexual selection will diverge from each other and the ancestral population. Also in the second scenario, relaxed selection is indistinguishable from genetic drift, but contributing loci would change less than expected under drift in populations with sexual selection. This implies that under the stabilizing selection scenario, the largest allele frequency differences are expected for neutral alleles, which are not contributing to sexual selection because they could diverge in both regimes. Loci involved in sexual selection can only drift in the regime with reduced sexual selection. Hence, with stabilizing selection, the contrast between populations with and without sexual selection is expected to be enriched for neutral loci, rather loci contributing to sexual selection.

An interesting case of stabilizing selection arises when sexual selection favors allele frequency differences in one direction, but pleiotropic effects act against these frequency changes. Alleles of the contributing loci reach an equilibrium frequency, which depends on the relative strength of these opposing effects. If sexual selection is removed by monogamy, only pleiotropic effects remain, which push allele frequencies in the same direction in all replicates. Such an effect was recently demonstrated by mixing two replicate populations, which evolved independently to the same new trait optimum, but did so with a different combination of contributing alleles (Christodoulaki et al. 2022).

Without making clear predictions about the expected allele frequency changes in this experiment, the authors contrasted populations with and without sexual selection to identify alleles with the most pronounced consistent allele frequency difference. This design is well-suited to identify directional selection with parallel selection signatures (i.e., selective sweeps), but the reliable identification of selection targets is highly contingent on the number of replicates and the duration of the experiment, in particular for small population sizes (Baldwin-Brown et al. 2014; Kofler and Schlötterer 2014). With only three replicates evolving for just 5 generations at a census size of 200, it appears very unlikely that the study of Wyer et al. (2023) has sufficient power to detect selection targets. Nevertheless, despite the low power of their experimental setting and against theoretical predictions, the authors identified more than 50,000 candidate SNPs.

## The Statistical Challenge

In the following, I will explain why Wyer et al. (2023) identified such a large number of candidate SNPs despite very limited statistical power. Wyer et al. (2023) used GLMM (and a liberal significance threshold of 10% after multiple testing), to identify SNPs with the most extreme allele frequency differences that consistently responded in all three replicates. The test performed by the authors is not suited to distinguish selection from genetic drift, as they did not fully account for stochastic sampling noise that occurs during five generations of experimental evolution in each of the regimes. In total, three levels of stochastic sampling need to be considered in statistical testing for selected loci. The first sampling step relates to genetic drift across multiple generations. The Wright–Fisher model is typically used to account for this sampling process. The next sampling step arises from the use of a subset of the total experimental population for sequencing, and the final sampling reflects which chromosomes are sequenced during Pool-Seq. These sampling properties are well understood, and software tools are available, which are designed to account for all three sampling steps (Spitzer et al. 2020). Because GLMM only accounts for the last sampling step and neglects the first two, a substantial excess of candidate SNPs (i.e., false positives) is expected from this analysis. Hence, the best explanation is that a large fraction of the identified candidate loci are false positives and do not contribute to a trait related to sexual selection. Note that loci contributing to a selected polygenic trait are unlikely to be among these candidates because no parallel selection response is expected for them (Barghi et al. 2019).

## Functional Testing

Consistent with widespread expectations of reviewers in the field, the authors did not rely on statistical testing only. Wyer et al. (2023) went an important step ahead and validated some candidate loci experimentally. Any experimental validation of candidate loci requires some a priori information about the affected trait (unless fitness consequences are tested). Wyer et al. (2023) assumed that insemination capacity is one of the traits subjected to stabilizing selection in the presence of sexual selection. For their functional validation, Wyer et al. (2023) focused on candidate loci, which introduced a premature stop codon, most likely resulting in a loss of function. Their functional testing aimed to replicate the influence of this mutation by RNA-mediated knockdown of the focal gene. Indeed, the knockdown of one of the four evaluated candidate genes reduced the insemination capacity from 89% to 55%. Despite the modest success rate of 25%, the authors considered the functional test as a confirmation of the candidates obtained in the genome scan.

In the following, I will discuss four reasons why this result should not be interpreted as a confirmation for an effect on sexual selection. First, the authors did not provide evidence that this insemination capacity actually evolved during their experiment. Only if populations with reduced sexual selection evolved a reduced insemination capacity during the five generations of experimental evolution, it is justified to use this trait in the empirical validation. Second, because high-level traits, such as insemination capacity, most likely have a complex genetic architecture, it is not sufficient to show that the knockdown of a candidate gene affects the trait: A randomly picked gene could also modify the phenotype of the focal trait. This problem has been recently empirically demonstrated. Zhang et al. (2021) used mutants to test two groups of genes for their effect on the focal trait. One set of genes was identified in a genome-wide association study (GWAS) screen, and the other group of genes consisted of randomly chosen genes that matched the characteristics of the candidate genes but were not detected in the GWAS screen. Remarkably, both groups resulted in a similar number of genes with an effect on the focal phenotype, which implies that functional testing by knockout/knockdown of candidate genes provides no additional support for the reliability of GWAS candidates. Only when the likelihood of a randomly selected gene to generate the same phenotypic effect is known it is possible to gauge the informativeness of the functional testing by gene knockdown. Third, it is important to consider the predicted direction of the phenotypic effect in individuals with dsRNA-mediated knockdown. The authors assume that insemination capacity is reduced in populations with reduced sexual selection. This can be achieved by either frequency increase or decrease of the premature stop codon in the populations evolving with reduced sexual selection. Hence, it is important to connect the direction of the evolutionary response of the focal locus with the expected phenotype. However, the authors do not provide their expectations for insemination capacity when the premature stop codon decreases during experimental evolution. In Figure 2, Wyer et al. (2023) show that in 5 out of 12 featured cases, the stop codon decreases in frequency in the populations with reduced sexual selection—does this imply that for those loci, a RNA-mediated knockdown would increase fertilization success? Finally, I question that the experimental design, as laid out by the authors, can result in different selection constraints on insemination capacity. The authors assume that females mate only with a single male, irrespective of whether only a single male or multiple males are present. Hence, although courtship-related traits may be under different selection in the two experimental regimes, selection on insemination capacity requires multiple mating. As the authors assume only single matings in their experiment, insemination capacity should not have evolved at all in this experiment. In the light of all these uncertainties, it becomes clear that insemination capacity is not the best trait to validate candidate genes for their role in sexual selection.

## Impact of Stabilizing Selection on Genetic Drift

Another prediction is that drift will be stronger in populations with reduced stabilizing selection compared with populations with stabilizing selection (note that for other forms of sexual selection, this prediction does not hold). Although the data of Wyer et al. (2023) fit this prediction, another prediction of stabilizing selection does not. In the presence of stabilizing selection, the affected loci are subject to reduced genetic drift, which in turn results in a larger effective population size than expected under neutrality (mostly due to linked selection). Under neutrality (i.e., relaxed selection), the census population size should match the estimated effective population size.

Genome-wide allele frequency changes provide accurate estimates of the effective population size in experimental evolution studies without selection (Jonas et al. 2016). Hence, based on the comparison of the effective population sizes in populations with and without sexual selection to the census size, it is possible to determine to what extent the populations match the pattern of relaxed (i.e., neutral evolution) or stabilizing selection. Interestingly, the estimated population size of populations from both regimes fitted neither the expectations for stabilizing selection nor the assumption of relaxed selection. The effective population size of populations with sexual selection is reduced to about ¼ of the census size. This implies either that stabilizing selection was not very effective or that other evolutionary forces increased the genome-wide allele frequency changes beyond the expectations of genetic drift under neutrality. The effective population size of replicates without sexual selection was only reduced by additional 50%. It is apparent that a rigorous quantitative assessment of this pattern would be necessary to provide support for the hypothesis of relaxed selective constraint, in particular as the reproductive success (probably due to differential fitness) must have been quite skewed in populations with and without sexual selection.

## Multiple Mating?

A closer look at the experimental procedures suggests another, probably more likely, explanation for the larger effective population sizes in replicates with sexual selection. The authors cultivated females either with five (sexual selection) or one (no sexual selection) males. Hence, multiple mating with different males in the sexual selection regime could easily explain the larger effective population size. In fact, visual observations have shown that the actual copulation time in *A. aegypti* is quite short (16 s on average) (Roth 1948). Additionally, studies involving cages with five males and five females have observed more than ten matings, implying that females engage in remating (Dieng et al. 2019). Perhaps the strongest evidence for multiple mating of *A. aegypti* comes from a microsatellite-based paternity study, which identified up to 34 different partners for males and females (Pimid et al. 2022). In the light of this overwhelming support for multiple mating in *A. aegypti*, the authors should have used paternity testing to rule out this simple, alternative scenario.

## Conclusion

The study of Wyer et al. (2023) demonstrates that functional testing with gene knockdown cannot compensate for insufficient statistical rigor because implicit assumptions about the focal phenotypic trait make the interpretation of such tests difficult. Hence, the application of rigorous statistical methods probably outweighs functional testing with RNA-mediated knockdown in most cases, an insight that clearly requires more awareness in the community. The study also sheds light on the fact that peer review is often not sufficient to identify conceptual and technical problems. This underscores the necessity for a collaborative community effort to detect such shortcomings. However, fostering such a culture demands a shift toward not only permitting but actively encouraging the publication of community feedback. *Current Biology* has chosen not to follow this spirit and desk rejected community feedback on Wyer et al. (2023) without peer review. The communication with *Current Biology* is provided as Supplementary material to this article.

## Supplementary Material

evad220_Supplementary_Data

## Data Availability

No new data were generated or analyzed in support of this article.
