# Supplementary material for: Unraveling the Molecular Basis of Stabilizing Selection by Experimental Evolution
Source: Genome Biol Evol. 2023 Dec 13;15(12):evad220. doi: 10.1093/gbe/evad220 (PMC10718812; doi:10.1093/gbe/evad220)
Supplement: evad220_Supplementary_Data [file evad220_supplementary_data.docx]

Dear editors,

a couple of weeks the study of Wyer et al. was published in Current Biology. After careful reading the paper, I have detected a series of mistakes, some methodological others conceptual in the paper, which make me conclude that the conclusions drawn by the authors are very likely to be incorrect. 

I have therefore written a response letter to the publication of Wyer et al. to highlight the problems and I would like to ask you to publish this response letter to raise the awareness that the results may not be accurate. 

many thanks for your consideration!


Christian Schlötterer

Dear Christian,

Thank you for your note, which I’ve discussed with my colleagues. While possibly fair comments on the study, I’m afraid we feel that they don’t rise to the level of requiring a formal comment in the journal. However, we would encourage you to contact the authors yourself to discuss these issues.

Best,

Cyrus

Cyrus Martin

Senior Scientific Editor, Current Biology

Dear Cyrus,

Many thanks for considering my response. I have to say, however, that I am extremely surprised about your decision.

1) the selection signature analysis of the authors is wrong and produces an excess of false positives

2) the transgenic test is based on a wrong phenotype

3) the key statement of single mating is not supported by the literature.

In other words everything reported in the study is an artificial based on incorrect statistical tests and wrong assumptions.

What else does it require to publish a cautionary note on a paper that should be retracted?

Many thanks for helping me to understand!

Chrisitan

I’m sorry Christian. We’ve considered and made a decision.

Cyrus Martin

Senior Scientific Editor, Current Biology
